# PNOC Expressed by B Cells in Cholangiocarcinoma Was Survival Related and LAIR2 Could Be a T Cell Exhaustion Biomarker in Tumor Microenvironment: Characterization of Immune Microenvironment Combining Single-Cell and Bulk Sequencing Technology

**DOI:** 10.3389/fimmu.2021.647209

**Published:** 2021-03-24

**Authors:** Zheng Chen, Mincheng Yu, Jiuliang Yan, Lei Guo, Bo Zhang, Shuang Liu, Jin Lei, Wentao Zhang, Binghai Zhou, Jie Gao, Zhangfu Yang, Xiaoqiang Li, Jian Zhou, Jia Fan, Qinghai Ye, Hui Li, Yongfeng Xu, Yongsheng Xiao

**Affiliations:** ^1^ Liver Cancer Institute, Zhongshan Hospital, Fudan University and Key Laboratory of Carcinogenesis and Cancer Invasion, Ministry of Education, Shanghai, China; ^2^ Neurosurgery Department of Zhongshan Hospital, Fudan University, Shanghai, China; ^3^ Department of Hepatobiliary and Pancreatic Surgery, The Second Affiliated Hospital of Nanchang University, Nanchang, China; ^4^ Department of Thoracic Surgery, Peking University Shenzhen Hospital, Shenzhen, China

**Keywords:** cholangiocarcinoma, immune infiltration, biomarker, single-cell sequencing technology, immunotherapy

## Abstract

**Background:**

Cholangiocarcinoma was a highly malignant liver cancer with poor prognosis, and immune infiltration status was considered an important factor in response to immunotherapy. In this investigation, we tried to locate immune infiltration related genes of cholangiocarcinoma through combination of bulk-sequencing and single-cell sequencing technology.

**Methods:**

Single sample gene set enrichment analysis was used to annotate immune infiltration status in datasets of TCGA CHOL, GSE32225, and GSE26566. Differentially expressed genes between high- and low-infiltrated groups in TCGA dataset were yielded and further compressed in other two datasets through backward stepwise regression in R environment. Single-cell sequencing data of GSE138709 was loaded by Seurat software and was used to examined the expression of infiltration-related gene set. Pathway changes in malignant cell populations were analyzed through scTPA web tool.

**Results:**

There were 43 genes differentially expressed between high- and low-immune infiltrated patients, and after further compression, PNOC and LAIR2 were significantly correlated with high immune infiltration status in cholangiocarcinoma. Through analysis of single-cell sequencing data, PNOC was mainly expressed by infiltrated B cells in tumor microenvironment, while LAIR2 was expressed by Treg cells and partial GZMB+ CD8 T cells, which were survival related and increased in tumor tissues. High B cell infiltration levels were related to better overall survival. Also, malignant cell populations demonstrated functionally different roles in tumor progression.

**Conclusion:**

PNOC and LAIR2 were biomarkers for immune infiltration evaluation in cholangiocarcinoma. PNOC, expressed by B cells, could predict better survival of patients, while LAIR2 was a potential marker for exhaustive T cell populations, correlating with worse survival of patients.

## Introduction

Cholangiocarcinoma (CCA) has long been deemed as a malignancy with poor prognosis in liver cancer. Patients conflicted by cholangiocarcinoma often are found in late stages, who were not candidates for surgery and seldom benefit from chemotherapy or comprehensive treatment (1, 2). Though blockade of programmed cell death receptor 1/programmed cell death receptor ligand 1 (PD1/PDL1) axis with mono-antibody, Pembrolizumab and Nivolumab, has shed light on partial patients, who showed high PDL1 expression in tumors, the overall treating efficacy in advanced CCA patients still needs further observation (3–6). Understanding tumor immune microenvironment (TIME) and infiltration status of CCA could better guide the clinical appliance of immunotherapy (7–9).

With the development of single-cell sequencing (scRNA-seq), investigators could further examine gene expression in individual cells and try to locate functional difference between different clinical phenotypes, especially in immune cells that have infiltrated the tumor (10–12). Characterization of CCA immune microenvironment is limited, so in this study to characterize immune cell components in TIME, we combined bulk sequencing data with scRNA-seq data, which could provide a better understanding of functional cell clusters related to disease severity. We found B cell infiltration levels in CCA TIME were related to patients’ overall survival (OS), and propronociceptin (PNOC), which was highly expressed by B cell populations in CCA, could be an independent indicator for better prognosis. Also, in CCA, leukocyte associated immunoglobulin like receptor 2 (LAIR2) was highly expressed by regulatory T cells (Tregs) and part of CD8+/GZMB+ T cells, which could be an indicator of exhaustive immune status in CCA patients. In addition, CCA cell sub-populations demonstrated heterogeneous metabolic and signal transduction activities, in which some CCA cells showed highly activated PD1/PDL1 axis signals, justifying the application of anti-PD1 combining therapy in CCA patients.

## Methods

### Datasets for Analysis and Derivation of Gene List

**Figure 1:**
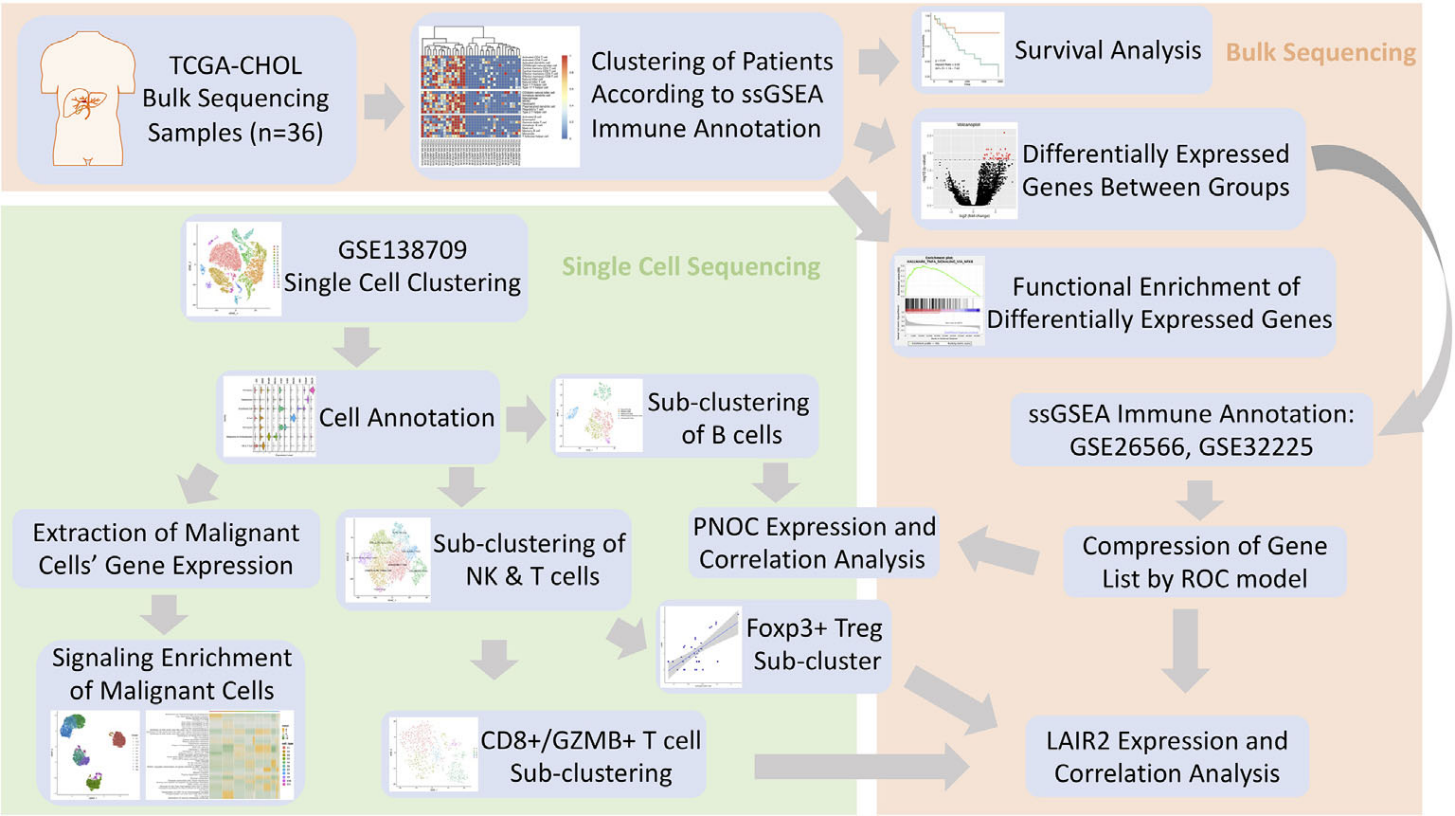
Flowchart of Investigation.

In this investigation, dataset of cholangiocarcinoma (CHOL) in database (n = 36) of the Cancer Genome Atlas (TCGA) (https://www.cancer.gov/about-nci/organization/ccg/research/structural-genomics/tcga) was used to analyze the differentially expressed genes between high- and low-immune infiltration groups (13). After searching Gene Expression Omnibus (GEO) database, data series with patient count over 100 were located, and the datasets with largest patient counts (GSE32225, n = 149; GSE26566, n = 104) were chosen for further immune infiltration classification (14, 15). Single cell sequencing data of five intrahepatic cholangiocarcinoma patients was procured from dataset of GSE138709 (16). The clinical information for patients’ cohorts were publicly accessible, which does not require additional endorsement from the local ethic committee. The immune meta gene list for 28 immune cell types were downloaded from TISBID database (http://cis.hku.hk/TISIDB/index.php) (17). Workflow of this Investigation was provided in **Figure 1**.

### Calculation of Immune Infiltration Scores in Bulk Sequencing Samples and Analysis of Differentially Expressed Genes Between Groups

In our analysis, single sample gene set enrichment analysis (ssGSEA) for immune infiltration annotation was performed to calculate respective immune infiltration scores of 28 immune cell types, which includes cell types of activated CD4 T cell, activated CD8 T cell, activated dendritic cell, CD56 bright natural killer (NK) cell, central memory CD4 T cell, central memory CD8 T cell, NK cell, NK T cell, type 1 T helper cell, type 17 T helper cell, CD56 dim NK cell, immature dendritic cell, macrophage, myeloid derived suppressive cell (MDSC), neutrophil, plasmacytoid dendritic cell, regulatory T cell (Treg), type 2 T helper cell, activated B cell, eosinophil, gamma delta T cell, immature B cell, mast cell, memory B cell, monocyte and T follicular helper cell (18). Differentially expressed genes between groups were analyzed using edgeR package, contrasting high- with low-immune infiltrated patients (19, 20).

### Gene Set Enrichment Analysis

Gene set enrichment analysis (GSEA) was used to demonstrate the altered pathways between patient groups in this study, using software of GSEA v4.1.0 (Broad Institute, Inc., Massachusetts Institute of Technology, and Regents of the University of California) (21). The annotation of changed pathways in this investigation was performed with hallmarks gene set (version: 7.2).

### Gene Ontology and Pathway Enrichment

DAVID database was used for gene ontology (GO) analysis, which included biological process, cellular compartment, and molecular function (https://david.ncifcrf.gov/summary.jsp) (22). The protein domains of differentially expressed genes between groups were also analyzed and downloaded from the database for demonstration. REACTOME database was also linked for annotation of significantly changed pathways between groups (www.reactome.org) (23).

### Single Cell Data Processing

For single cell sequencing analysis, raw data for GSE138709 were downloaded from portal website, and package of Seurat was used to process data in R (version: 4.0.3) with R studio (version: 1.3.1903) (24–26). The raw data GSE138709 were loaded with Seurat, and cells were filtered with the criteria of >20% mitochondria related genes or more than 6,000 genes expressed. A total of 32,627 cells were included for further analysis, and variable features of each sample were analyzed after normalization. Then we used Seurat function of FindIntegrateionAnchors to merged sample files with common anchors among variables (dims=1:20, k.filter=30) (26). Merged data of cells were clustered into 15 cell populations using function of FindClusters (resolution = 0.3). Respective reduction of cell clustering, including UMAP, TSNE, and PCA, were performed. For cell population annotation, we used the signatures chosen in the original publication (16). For NK and T cell cluster, signatures of CD7, FGFBP2, KLRF1, CD2, CD3D, and CD3E were chosen for annotation. For malignancy and cholangiocyte, signatures of EPCAM, KRT19, KRT7, FXYD2, TM4SF4, and ANXA4 were chosen. For monocytes, CD14 and CD11C were chosen for annotation. For B cell cluster, CD79A, MS4A1 were chosen. For endothelial cells, signatures of ENG and VWF were chosen for annotation. For hepatocytes, APOC3, FABP1, and APOA1 were chosen for annotation. And for fibroblasts, ACTA2 and COL1A2 were chosen for demonstration.

### Analysis of Pathway Changes in Malignant Cholangiocarcinoma Cells

To compute and analyze pathway scores in malignant cholangiocarcinoma cells, we used scTPA, which is a web tool for single-cell analysis of activated pathways (http://sctpa.bio-data.cn:8080/index.html) (27, 28). The malignant cell expression matrix was extracted by sample origins in malignancy and cholangiocyte cluster, and then expression matrix was uploaded online. Analyzed results were downloaded for further analysis and demonstration.

### Correlation Between Specific Genes and Immune Infiltration Scores

TIMER 2.0 web tool (http://timer.cistrome.org) was used for correlation of gene expression with immune cell infiltration scores, which included scores calculated by CIBERSORT and MCP-counter methods (29–31). Scores of TCGA CHOL sequencing data calculated by other infiltration estimating methods were also downloaded from website for analysis.

### Correlation Between Specific Gene Markers

Database GEPIA (http://gepia.cancer-pku.cn) was used for correlation analysis between PNOC, LAIR2, and a series of immune regulators in bulk sequencing data of CHOL and hepatocellular carcinoma (LIHC) in TCGA database (32).

### Survival Analysis of Genes in Outside Database

Survival analysis of specific genes was performed in outside database, KMplotter, which is an integrated portal for tumor survival analysis, combining genomics data of microarray with clinical information (33).

### Statistics

Survival analysis in this investigation was performed with R packages survival and survminer in R environment, which were used to find the best cutoff values for survival comparison between groups (34). Package of pheatmap was used to construct heat maps (35). Dot plots for correlation analysis and bar plots for GO analysis were generated by packages ggplot2, using spearman correlation test, and GOplot respectively (36, 37). A generalized linear model (GLM) in R was used for prediction of immune infiltration status, using differentially expressed genes, and then stepwise algorithm (backward) was used to choose the appropriate model by an information criterion (AIC) extracted from formerly fitted model (AIC= −2*log L + k* edf; L: likelihood; edf: the equivalent degrees of freedom). Receiver operating characteristics (ROC) were examined using package plotROC. P value under 0.05 was considered significant.

## Results

### Cholangiocarcinoma Patients in TCGA Dataset Were Clustered Into High- and Low-Immune Infiltrated Groups With Different Prognosis

**Figure 2:**
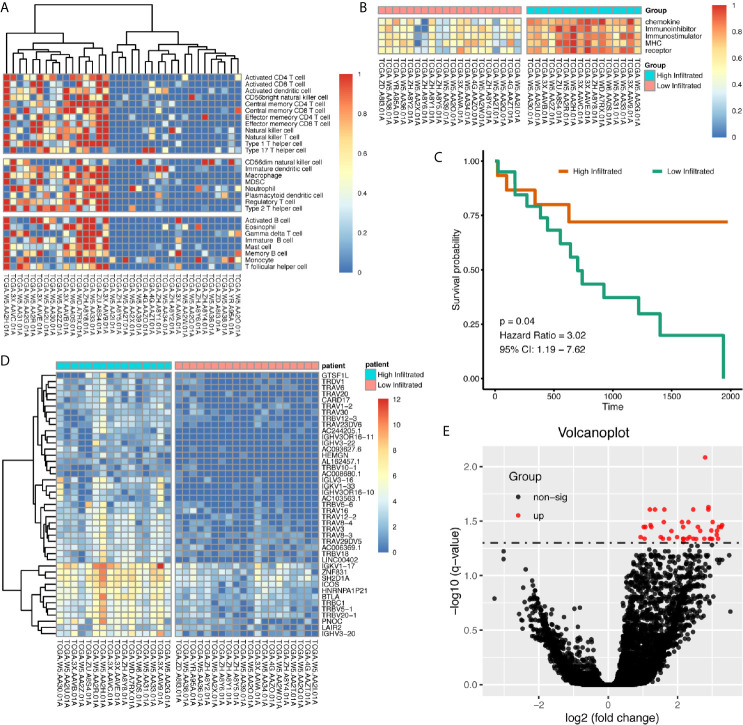
Patients with cholangiocarcinoma were divided into differentially immune infiltrated groups with different prognosis. **(A)** Clustering of CCA patients according to immune infiltration status calculated by ssGSEA method. **(B)** Whole scores of chemokine, chemokine receptor, immune stimulator, immune inhibitor, and MHC expression levels between groups. **(C)** Survival difference between high- and low-immune infiltrated cholangiocarcinoma patients. **(D, E)** Differentially expressed genes between high- and low-immune infiltrated patients.

Using immune gene list for 28 immune infiltrating cell populations, we generated scores for each immune cell type. After clustering cholangiocarcinoma patients according to the calculated scores, we found there was a clearly different immune status between groups (**Figure 2A**). We also used gene lists for immune stimulators, inhibitors, MHC molecules, chemokine, and chemokine receptors to calculate the corresponding scores, and in high-immune infiltration patients, expression levels for those genes were much higher (**Figure 2B**). Patients with high immune infiltration showed better prognosis (**Figure 2C**).

### Differentially Expressed Genes Between High- and Low-Infiltrated Patients Were Mainly About Immune Functions, and Inflammatory Signals Were Highly Enriched in High-Immune Infiltrated Patients

**Figure 3:**
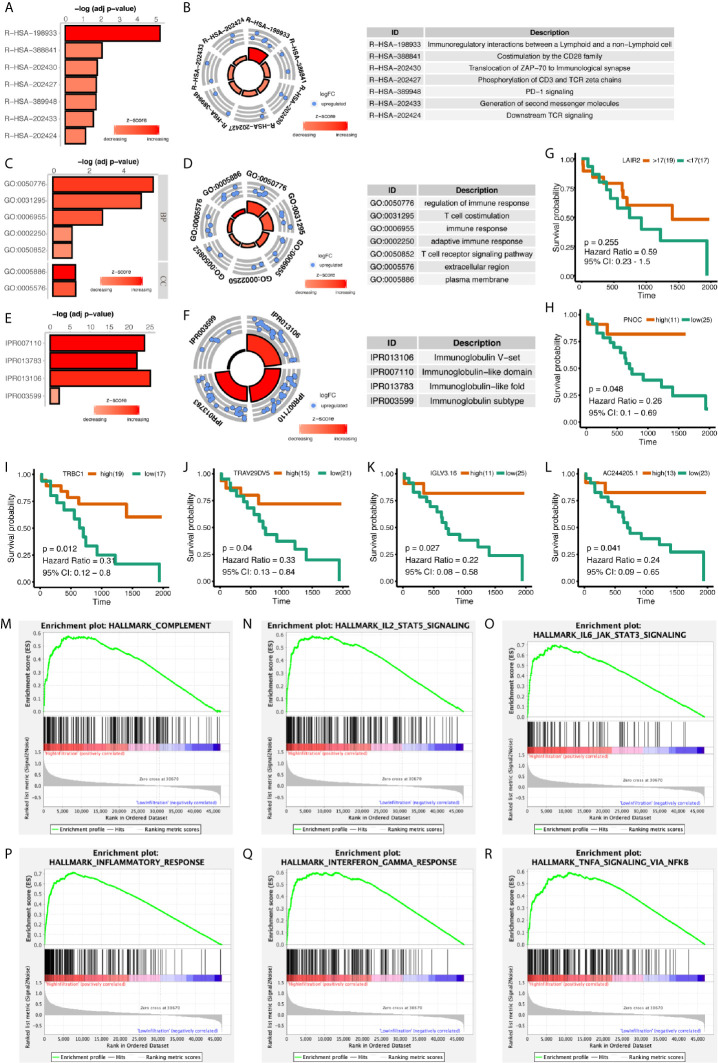
Functional Enrichment of Differentially Expressed Genes Between High- and Low-Immune Infiltration Groups. **(A, B)** Pathway enrichment of differentially expressed genes in REACTOME database. **(C, D)** Gene ontology enrichment of differentially expressed genes. **(E, F)** Protein function enrichment of differentially expressed genes. **(G–L)** Among differentially expressed genes, PNOC, TRBC1, TRAV29DV5, IGLV3.16, and AC244205.1 were significantly correlated with CCA patients’ overall survival, while LAIR2 did not achieve significance. **(M–R)** Signatures of complement pathway, IL2-STAT5 pathway, IL6-Jak-STAT3 pathway, inflammatory response pathway, interferon-gamma response pathway, and TNF *via* NFKB pathway were highly enriched in high-immune infiltrated patients.

Differentially expressed genes between high- and low-infiltrated groups were analyzed, and only a set of 43 genes were up-regulated in high-infiltrated patients (**Figures 2D, E**) Pathway enrichment showed the up-regulated gene set was mainly about inflammatory signals, immune stimulation, and PD1 axis (**Figures 3A, B**). Gene ontology enrichment for 43 gene set showed those genes were involved in the process of adaptive immune responses and T cell signaling (**Figures 3C, D**). The protein functional enrichment showed most of the 43 genes were immunoglobulins (**Figures 3E, F**). Among those genes that were significantly survival-related, all could indicate better overall survival with higher expression (**Figures 3G–L**). Further gene set enrichment analysis between groups showed hallmarks of complement signaling, IL2/STAT5 signaling, IL6/JAK/STAT3 signaling, inflammatory response signaling, interferon gamma signaling, and TNFA signaling *via* NFKB were highly enriched (**Figures 3M–R**).

### Several Genes Were Associated With Immune Infiltration Status by Stepwise Regression Model

We further calculated immune infiltration scores for datasets of GSE26566 and GSE32225, and after clustering patients into high- and low-infiltration groups, we used backward stepwise regression model to compress the 43 gene set in prediction of immune infiltration status in the two datasets respectively (**Table 1**). In both models (GSE26566: infiltration score = 6.846 − 0.053*SH2D1A – 0.061*PNOC – 0.021*LAIR2; GSE32225: infiltration score = −1.690 + 0.014*SH2D1A – 0.007*LAIR2 – 0.010*ICOS + 0.019*HEMGN + 0.012*GTSF1L), LAIR2 were related to high-immune infiltration status (**Supplementary Figure 4**).

**Table 1:** Stepwise Regression Model for Compression of Immune Infiltration Related Genes.

Datasets		Estimate	Std. Error	z value	Pr(>|z|)
GSE26566	(Intercept)	6.84600446	1.44844569	4.72644885	2.28E-06
	SH2D1A	−0.0527032	0.01226927	−4.2955398	1.74E-05
	PNOC	−0.0612851	0.04286357	−1.4297708	0.15278282
	LAIR2	−0.0205321	0.00803995	−2.5537545	0.01065684
GSE32225	(Intercept)	−1.6900303	1.95226226	−0.8656779	0.38666682
	SH2D1A	0.01434228	0.01042714	1.37547498	0.16898424
	LAIR2	−0.0074253	0.00197153	−3.7662633	0.00016571
	ICOS	−0.0098082	0.00372504	−2.6330564	0.00846203
	HEMGN	0.0187238	0.00680099	2.75309954	0.00590339
	GTSF1L	0.0122422	0.00485591	2.52109161	0.01169914

### Further Demonstration of CCA Tumor Microenvironment Showed PNOC Was Mainly Expressed by B Cell, Which Was Also an Indicator for Better Prognosis

**Figure 4:**
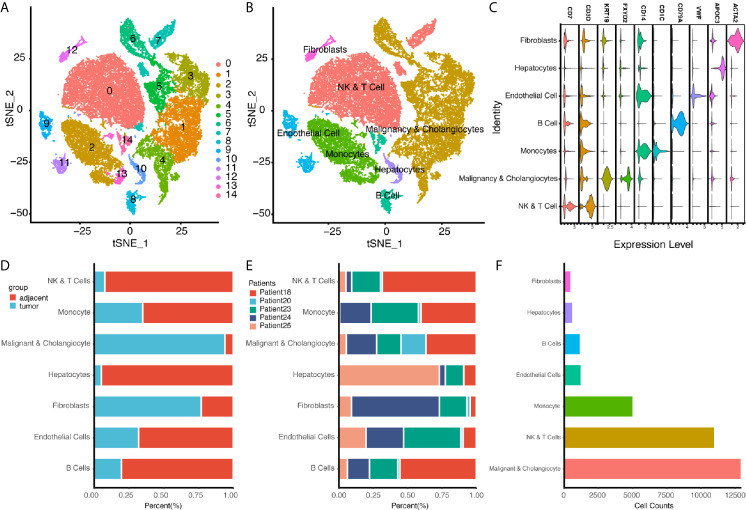
Single Cell Atlas of CCA Patients According to Dataset GSE138709. **(A, B)** Cell clusters for GSE138709 of five CCA patients. **(C)** Cell markers for clusters’ annotation. **(D)** Portions of adjacent and tumor tissues in different cell clusters. **(E)** Patient portions in different cell clusters. **(F)** Numbers for cell clusters in dataset after filtration.

In addition to bulk sequencing analysis, we analyzed the immune microenvironment of intrahepatic cholangiocarcinoma with single cell sequencing dataset GSE138709. We further clustered cell populations into 15 clusters, and using genes CD7, CD3D, KRT19, FXYD2, CD14, CD1C, CD79A, VWF, APOC3, and ACTA2, we classified 15 cell clusters into 7 cell populations, which were fibroblasts, NK and T cells, malignancy and cholangiocytes, endothelial cells, monocytes, hepatocytes, and B cells (**Figures 4A–C**). Proportions of cells in different tissue types showed most cells in malignancy and cholangiocyte cluster were from tumor samples, while most cells in NK&T cell cluster were form adjacent samples. Also, a high portion of fibroblasts were also seen in tumor samples (**Figures 4D–F**).

**Figure 5:**
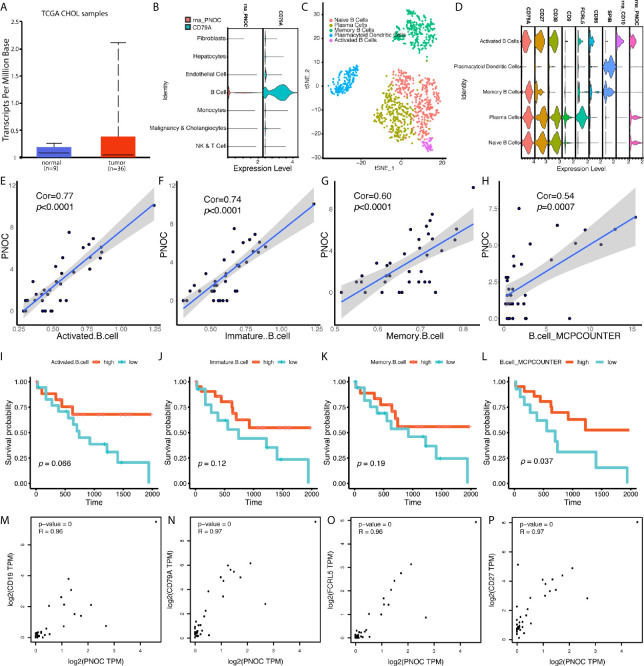
PNOC Was Highly Expressed by B Cell Populations in CCA, and B Cell Infiltration Levels in CCA Indicated Better Overall Survival. **(A)** PNOC was highly expressed in CCA tumors in TCGA database, though significant difference was not achieved. **(B)** PNOC was mainly expressed by B cells in single cell levels. **(C)** Further cluster of B cell populations. **(D)** Markers for sub B cell populations. **(E–H)** Correlation between PNOC expression and scores for activated CD8+ T cell, immature B cell, memory B cell, and whole B cell calculated by ssGSEA and MCPcounter methods. **(I–L)** B cell infiltration levels for activated CD8+ T cell, immature B cell, memory B cell, and whole B cell calculated by ssGSEA and MCPcounter methods were correlated with CCA patients’ overall survival. **(M–P)** Correlation between CD19, CD79A, FCRL5, CD27, and PNOC in CHOL bulk sequencing samples from GEPIA database.

We found PNOC was highly expressed in tumors, though the difference between normal and tumor tissues was not significant (**Figure 5A**). We further examined genes’ expression, selected by stepwise regression models, in single cell populations, and PNOC was mainly expressed by B cell cluster. After sub-clustering, activated B cells, plasma cells, and naive B cells all showed expression of PNOC (**Figures 5B–D**). We further examined the correlations between PNOC and scores for B cell infiltration in TCGA CHOL samples, calculated by ssGSEA and MCPcounter methods, and results showed PNOC was highly correlated with B cells (**Figures 5E–H**). Also, we found high B cell infiltration scores in cholangiocarcinoma were related to better prognosis, though survival benefits of high immature B cell and memory B cell scores were not significant due to small sample size (**Figures 5I–L**). We further used database GEPIA to examine B cell markers’ correlation with PNOC, and results showed PNOC was highly correlated with CD19, CD79A, CD27, and FCRL5 in bulk sequencing data of CHOL (Coefficients >0.95) (**Figures 5M–P**).

### LAIR2 Was Up-regulated in CCA Samples, Which Was Mainly Expressed by Regulatory T Cells and a Subset of CD8+/GZMB+ T Cells

**Figure 6:**
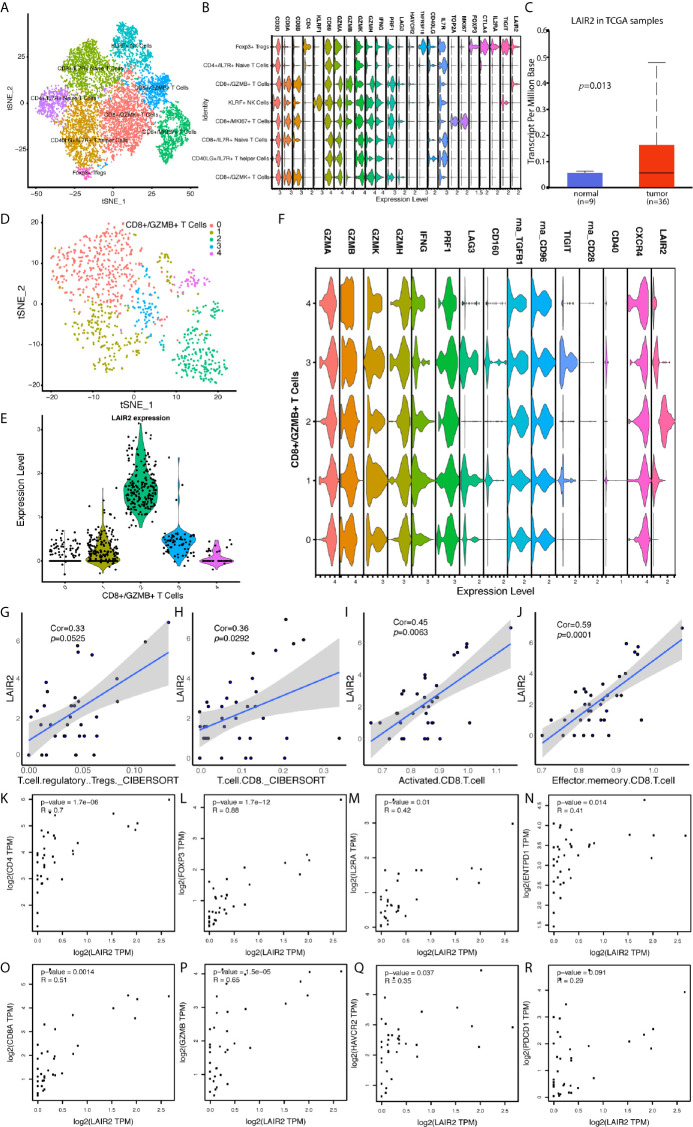
LAIR2 Was Highly Expressed by Regulatory T Cells and CD8+/GZMB+ T Cell Subset. **(A)** TSNE reduction for demonstration of NK and T cell atlas. **(B)** Markers for sub T and NK cell populations. **(C)** LAIR2 expression levels between CCA tumor and normal tissues in TCGA database. **(D)** Further cluster of CD8+/GZMB+ T cells. **(E)** LAIR2 expression in further clustered CD8+/GZMB+ T cell sub-populations. **(F)** Functional markers’ expression levels between further clustered CD8+/GZMB+ T cell sub-populations. **(G–J)** Correlation between LAIR2 expression and scores for regulatory T cell and CD8+ T cell calculated by CIBERSORT or ssGSEA method. **(K–N)** Correlation between LAIR2 and Treg markers [CD4, FOXP3, IL2RA (CD25), and ENTPD1 (CD39)] in CHOL bulk sequencing data from GEPIA database. **(O–R)** Correlation between CD8A, GZMB, HAVCR2 (TIM3), PDCD1 (PD1), and LAIR2 in CHOL bulk sequencing data.

**Figure 7:**
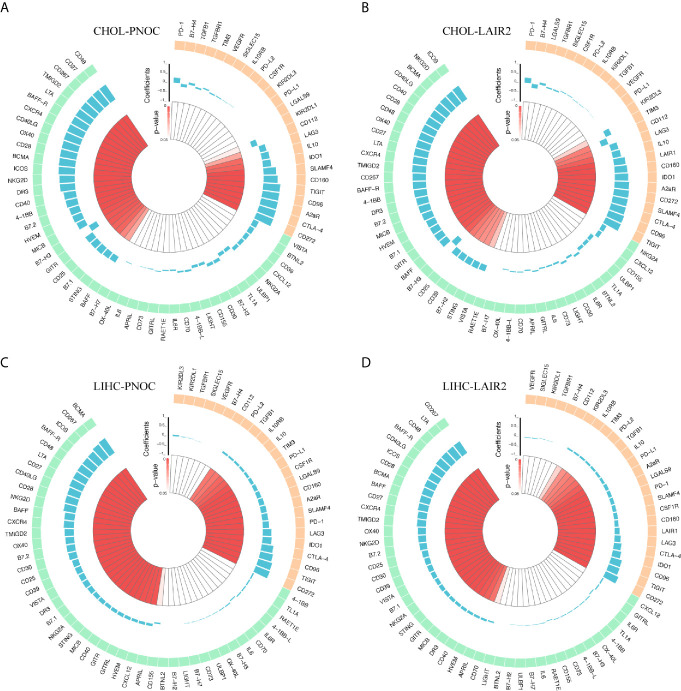
Correlation Between LAIR2, PNOC, and Acknowledged Immune Checkpoints in TCGA CHOL and LIHC Datasets. **(A)** Correlation between expression of PNOC and immune regulators in CHOL dataset. **(B)** Correlation between expression of LAIR2 and immune regulators in CHOL dataset. **(C)** Correlation between expression of PNOC and immune regulators in LIHC dataset. **(D)** Correlation between expression of LAIR2 and immune regulators in LIHC dataset. (Immune inhibitors were marked with light orange, while immune stimulators were marked with light green. P values over 0.05 were not significant and were marked with white color.)

We further divided NK and T cells populations into sub-clusters, and LAIR2 was found to be expressed by Foxp3+ regulatory T cell and CD8+/GZMB+ T cell clusters (**Figures 6A, B**). In TCGA CHOL samples, LAIR2 expression was increased in tumor samples (**Figure 6C**). After further clustering of CD8+/GZMB+ T cells into five sub-clusters, we found four of them showed expression of LAIR2, in which sub-cluster 2 demonstrated higher expression (**Figures 6D, E**). In addition, in comparison to immune stimulators (CD28, CD40), immune inhibitors (TGFB1, CD96, TIGIT, and LAG3) were highly expressed by all those sub-clusters, especially clusters 1 and 3 (**Figure 6F**). We further correlated LAIR2 expression with Treg scores and CD8+ T cell scores in TCGA CHOL samples, calculated by CIBERSORT and ssGSEA methods, and results showed LAIR2 was correlated to those cell populations (**Figures 6G–J**). We correlated LAIR2 with Treg cell markers (CD4, FOXP3, CD25, and CD39) and CD8+ T cell markers (CD8A, GZMB, TIM3, and PD1) in CHOL dataset, which all demonstrated high coefficients. Though the correlation between PD1 and LAIR2 was obvious, the corresponding coefficient did not achieve significance (**Figures 6O–R**). Considering immune regulation was performed through cooperation of immune regulators, we additionally analyzed the correlation between LAIR2, PNOC, and commonly acknowledged immune regulators, and results showed both LAIR2 and PNOC were significantly highly correlated with a bunch of immune inhibitors and stimulators in TCGA CHOL sequencing samples (**Figure 7**).

### CCA Cells Demonstrated Heterogeneous Pathway Changes in Single Cell Level, Which Indicated Functional Variance and Malignant Potentials of Different Cancer Cell Clusters

We further extracted malignancy expression matrix from chonlangiocytes’ expression and calculated REACTOME pathway scores for each cell. After clustering of malignant cells according to calculated scores, cells were clustered into 11 populations (**Supplementary Figures 2A, B**). Of notice, clusters 11, 2, 7, 8, and 9 demonstrated highly malignant traits with high expression of signatures in cell mitotic cycle, IL1 signaling, PD1 signaling, and PI3K signaling (**Supplementary Figure 2C**).

## Discussion

In our analysis, we used bulk sequencing data of cholangiocarcinoma patients in TCGA database to calculate the immune infiltration scores of different immune cell populations, and then we compared expression difference between groups, locating immune infiltration highly associated genes; we found PNOC was mainly expressed by infiltrated B cells, which was survival related, while LAIR2 was mainly expressed by Tregs and partial CD8+/GZMB+ T cells, indicating exhaustive immune status of T cells.

Prepronociceptin (PNOC) was formerly reported to be a pre-protein for a series of products, which act as pain regulators in signal transduction (38). Recent study showed PNOC is involved in long-term opioid response, alcoholic states, and inflammation process (39–44). In cancer investigations, PNOC was related to high inflammatory status and oxidative stress in C6 glioma cells, which also was highly up-regulated in pediatric brainstem ganglioglioma tissues and epithelial ovarian cancer, and in analysis of high-risk gastrointestinal stromal tumor, PNOC was reported as a prognostic biomarker (45–48). In a study of mRNA and microRNA network in colorectal cancer, PNOC and its targeting microRNAs were also prognostic markers for evaluation of patients (49). In our analysis, PNOC was up-regulated in cholangiocarcinoma samples, though the difference didn’t achieve significance. Low expression of PNOC may explain why only one model of GEO datasets included PNOC for prediction of high-immune infiltration; further analysis showed, PNOC was highly expressed by B cell populations in TIME. Expression of PNOC and infiltrating levels of B cell populations in CHOL were both survival-related, and in our analysis, differentially expressed immune genes between high- and low-immune infiltration groups were mainly immunoglobulins, indicating B cell infiltration was crucial in humoral anti-tumor responses. We also examined the prognostic values of PNOC in hepatocellular carcinoma, and we found patients with high PNOC expression also had better overall survival, indicating PNOC could be an independent biomarker for patients’ evaluation (**Supplementary Figure 3B**). High PNOC expression could predict highly infiltrated TIME. The specific roles of PNOC, expressed by B cells in TIME regulation, still need further experiments to illustrate.

Leukocyte associated immunoglobulin like receptor 2 (LAIR2) was previously reported as a close member to leukocyte associated immunoglobulin like receptor 1 (LAIR1), which is deemed as an immune inhibitor expressed by immune cells (50–52). According to publications, expression of LAIR1 was detected in various immune cell populations, and it has immune receptor tyrosine-based inhibition motif (ITIM), recruiting SHP-1, SHP-2, and Src kinase after phosphorylation (53, 54). Collagen in tumor matrix and damaged tissues is a common ligand for LAIR1 in broad spectrum, inhibiting immune cell functions after ligation, while LAIR2 was found to be a soluble protein with similar extracellular domain, which could block LAIR1 binding by competing ligands (55–58). Former studies also showed in autoimmune diseases, expression of LAIR2 was increased, and genetic single nucleotide polymorphism of LAIR2 was related to susceptibility of autoimmune diseases (59–62). The knowledge of LAIR2 in TIME regulation is limited, however, LAIR2 could interfere platelet activation and adhesion, and secreted LAIR2 could inhibit classical and lectin pathways of complement system in killing pathogens (58, 63). Overexpression of LAIR2 in lung cancer could increase immune infiltration levels and rescue exhaustive CD8+ T cells’ function (58). In our analysis, the mRNA expression of LAIR2 in Tregs and partial CD8+/GZMB+ T cells, in comparison to LAIR1, which was widely expressed by various cell populations, could be an indicator of exhaustive immune status. Former studies showed LAIR1 was expressed by macrophages, dendritic cells, as well as other CD14+ cells in inflammation, and scientists hypothesized LAIR1 could be both a threshold receptor and negative feedback receptor (64, 65). In our analysis, LAIR1 was not related to immune infiltration status in CHOL, and we believed mRNA expression of LAIR2 may be increased to offset LAIR1’s function in feedback loop, highlighting the role of baseline LAIR2 expression. Though LAIR2 in bulk sequencing data of CHOL was not survival-related, high LAIR2 expression in LIHC could indicate worse prognosis (**Supplementary Figure 3A**). We further examined coefficients for correlations between LAIR2 and other acknowledged immune regulators, such as CD28, LAG3, CD40, CXCR4, and TIGIT, in CHOL and LIHC datasets respectively, most of which achieved significance with high coefficients.

In addition, we analyzed the pathway changes in intrahepatic cholangiocarcinoma cell populations, finding functionally heterogeneous cancer cell clusters. Those malignant cells were clustered into 11 populations, in which several clusters showed high self-replication potentials, while others showed activated PI3K signal cascade through FGFR interaction. Also, cluster 2 and cluster 11 cells showed immune evasion potentials by increasing human lymphocyte associated antigens, and in cluster 7, expression of PDL1 (CD274) was increased. These functionally different cell populations in tumor justify the need of combining immune therapy in cholangiocarcinoma, and PNOC and LAIR2 could be clinical biomarkers for patient evaluation before immune therapy, predicting patients’ survival and tumor immune infiltration accordingly.

There are some limitations of our study. First, though we combined bulk sequencing and single cell sequencing data to characterize TIME of CHOL, protein expression were not examined, and further experiments should be conducted for confirmation. Second, immune regulating roles of PNOC, expressed by B cells, and LAIR2, expressed by Tregs and partial CD8+/GZMB+ T cells, in TIME are still in mist, which shall be further investigated.

## Conclusion

High B cell infiltration level could indicate better prognosis in CCA. PNOC was mainly expressed by B cells in TIME and could be an independent indicator for better prognosis. LAIR2 was mainly expressed by Treg and partial CD8+/GZMB T cells, which could be an indicator for exhaustive T cell populations in TME. Both PNOC and LAIR2 were correlated with high immune infiltration levels in CCA patients.

## Data Availability Statement

Publicly available datasets were analyzed in this study. This data can be found here: CHOL and LIHC in TCGA database: https://www.cancer.gov/about-nci/organization/ccg/research/structural-genomics/tcga: GSE32225: https://www.ncbi.nlm.nih.gov/geo/query/acc.cgi?acc=GSE32225, GSE26566: https://www.ncbi.nlm.nih.gov/geo/query/acc.cgi?acc=GSE26566, GSE138709: https://www.ncbi.nlm.nih.gov/geo/query/acc.cgi?acc=GSE138709.

## Ethics Statement

Ethical review and approval was not required for the study on human participants in accordance with the local legislation and institutional requirements. Written informed consent for participation was not required for this study in accordance with the national legislation and the institutional requirements.

## Author Contributions

ZC, MY, JY, LG, BZ, and SL contributed to designing and analyzing process of the investigation, drafting the manuscript afterwards. JL, WZ, BHZ, JG, ZY, and XL helped to collect data and perform analysis correspondingly. JZ, JF, QY, HL, YFX, and YSX reviewed the whole manuscript, adapting the manuscript for final submission. All authors contributed to the article and approved the submitted version.

## Funding

This research was funded by National Nature Science Foundation of China (81871924, 81572844, 81572301, 81802893, 81502487, 81972829), Natural Science Foundation of Guangdong Province of China (Grant Nos. 2017A030310641, 2018A030313762), and Foundation of Shanghai Municipal Commission of Health and Family Planning (20174Y0072).

## Conflict of Interest

The authors declare that the research was conducted in the absence of any commercial or financial relationships that could be construed as a potential conflict of interest.

## Supplementary Material

Supplementary Table 1 Immune Infiltration Classification of Patients in Datasets of GSE26566 and GSE32225.

Supplementary Table 2 Coefficients for LAIR2 and PNOC in Association With Treg, CD8+ T cells and B cells in Bulk Sequencing Data of CCA in TCGA Database Calculated by TIMER 2.0.

Supplementary Table 3 Clinical information for CHOL bulk sequencing samples in TCGA database.

Supplementary Figure 1 Expression of Immune Inhibitors and Stimulators in High- and Low-Immune Infiltration Patients.

Supplementary Figure 2 Heterogeneous Pathway Changes in Intrahepatic Cholangiocarcinoma Cells Demonstrated Different Functional Status of Sub-cell Populations. **(A)** Heatmap for pathway scores of different cholangiocarcinoma cell sub-populations. **(B)** UMAP reduction for demonstration of cholangiocarcinoma cell sub-populations. **(C)** Heatmap for genes’ expression in PD1 signaling, Cell Cycle Mitotic signaling, IL-1 signaling, and PI3K-FGFR signaling between groups.

Supplementary Figure 3 Both of PNOC and LAIR2 Were Related to Overall Survival of HCC Patients. **(A)** High expression of LAIR2 indicated worse survival in HCC patients. **(B)** High expression of PNOC indicated better survival in HCC patients.

Supplementary Figure 4 ROC Plots for Immune Infiltration Models’ Evaluation. **(A)** ROC curves for regression model of immune infiltration score and each infiltration-related gene in dataset of GSE26566. **(B)** ROC curves for regression model of immune infiltration score and each infiltration-related gene in dataset of GSE32225 (AUC, area under curve).
