# Supplementary figures and images for: PNOC Expressed by B Cells in Cholangiocarcinoma Was Survival Related and LAIR2 Could Be a T Cell Exhaustion Biomarker in Tumor Microenvironment: Characterization of Immune Microenvironment Combining Single-Cell and Bulk Sequencing Technology
Source: Front Immunol. 2021 Mar 24;12:647209. doi: 10.3389/fimmu.2021.647209 (PMC8024580; doi:10.3389/fimmu.2021.647209)

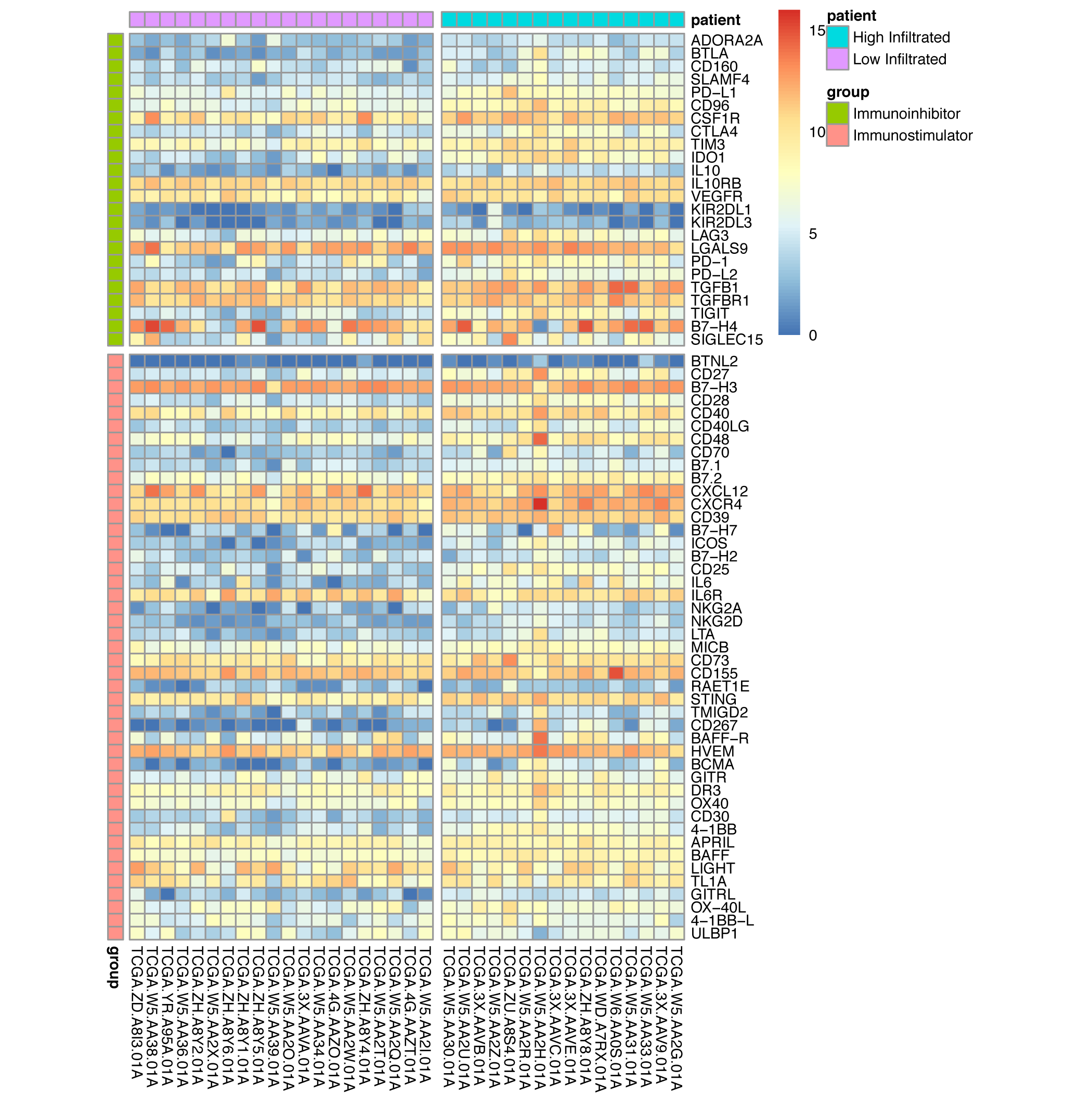

Supplement: Supplementary Figure 1 — Expression of Immune Inhibitors and Stimulators in High- and Low-Immune Infiltration Patients. [file Image_1.tif]

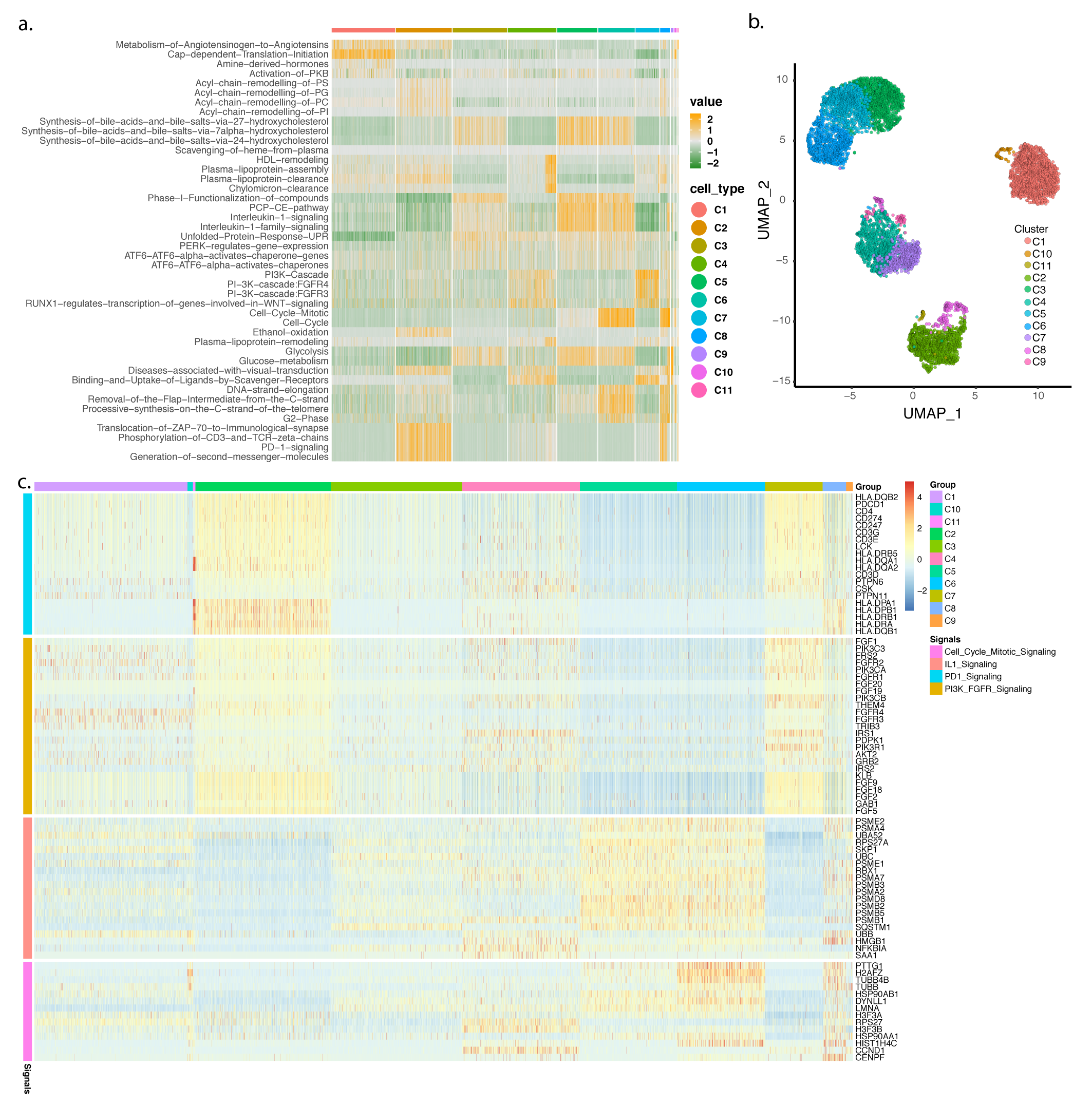

Supplement: Supplementary Figure 2 — Heterogeneous Pathway Changes in Intrahepatic Cholangiocarcinoma Cells Demonstrated Different Functional Status of Sub-cell Populations. (A) Heatmap for pathway scores of different cholangiocarcinoma cell sub-populations. (B) UMAP reduction for demonstration of cholangiocarcinoma cell sub-populations. (C) Heatmap for genes’ expression in PD1 signaling, Cell Cycle Mitotic signaling, IL-1 signaling, and PI3K-FGFR signaling between groups. [file Image_2.tif]

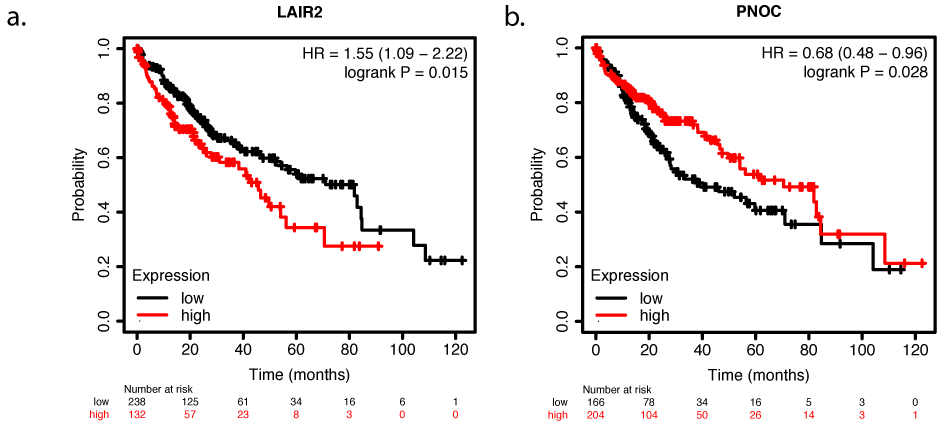

Supplement: Supplementary Figure 3 — Both of PNOC and LAIR2 Were Related to Overall Survival of HCC Patients. (A) High expression of LAIR2 indicated worse survival in HCC patients. (B) High expression of PNOC indicated better survival in HCC patients. [file Image_3.tif]

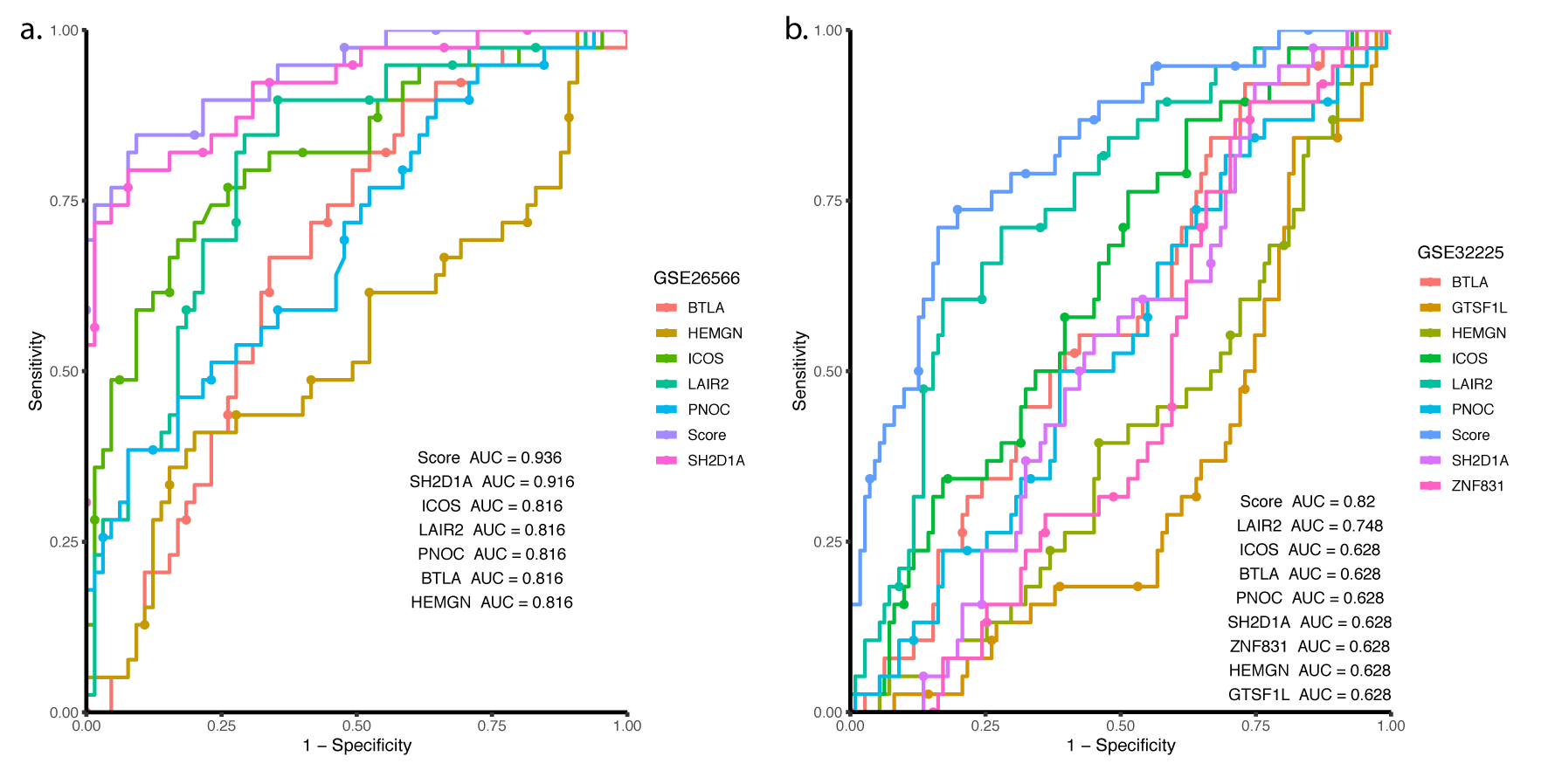

Supplement: Supplementary Figure 4 — ROC Plots for Immune Infiltration Models’ Evaluation. (A) ROC curves for regression model of immune infiltration score and each infiltration-related gene in dataset of GSE26566. (B) ROC curves for regression model of immune infiltration score and each infiltration-related gene in dataset of GSE32225 (AUC, area under curve). [file Image_4.tif]
